# Primary spinal oligodendroglioma with intracranial extension: a case report

**DOI:** 10.1097/MS9.0000000000002099

**Published:** 2024-04-29

**Authors:** George Bashour, Nahar Ismaiel, Yousef Ebrahim, Manar Ibrahim, Tala Deeb, Karam Khatab, Mohammad S. Ali, Zuheir Alshehabi

**Affiliations:** a Cancer Research Center; b Department of Pathology; c Department of Neurosurgery, Tishreen University Hospital; d Faculty of Medicine, Tishreen University, Latakia, Syria

**Keywords:** intracranial extension, intramedullary spinal tumors, oligodendroglioma

## Abstract

**Introduction:**

Primary spinal cord oligodendrogliomas (PSO) are sporadic tumors that arise from oligodendrocytes in the central nervous system (CNS). They can affect adults and children and make up about 2% of all intramedullary (IM) spinal tumors. Here, the authors present the second case in the literature of a primary spinal oligodendroglioma with intracranial extension.

**Presentation:**

A 28-year-old right-handed female presented to our emergency room severely malaised with left-sided hemiparesis, numbness, tingling, and urinary retention with positive Babinski and negative Hoffmann. MRI showed a widespread heterogeneous mass extending from the medulla to C7 with syringomyelia inferior to the mass. The mass was removed surgically, and her neurological condition improved rapidly. The gross, pathological exams, and immunohistochemistry confirmed the diagnosis of oligodendroglioma.

**Discussion:**

Up until 2017, there have been 60 documented cases of PSO in the literature and we have found two more cases in our search between 2017 and 2023. Also, there has been only one case recorded with an intracranial extension, making our case the 63rd PSO case and the second one with cranial extension.

**Conclusion:**

The golden standard for imaging is MRI. Surgical excision is the main treatment in the literature. Single-stage laminectomy showed promising results and surgical resection was the critical intervention to which the patient responded. This matches what was stated in the literature that surgery is the primary mode of treatment in PSO patients.

## Introduction

Highlights

- Primary spinal cord oligodendrogliomas are extremely rare tumors that arise from oligodendrocytes present in the spinal cord.
- Only 62 cases have been reported in the literature so far.
- Intracranial extension has been reported only in one other case.
- We report the second case in the literature of intracranial extension.
- Surgery is the main treatment whether it is done on one or two stages.

Primary spinal cord oligodendrogliomas (PSOs) are sporadic tumors that arise from oligodendrocytes present in the spinal cord. This type of tumor is usually detected in the cerebral hemisphere, but both primary and drop metastasis spinal oligodendroglioma are atypical clinical presentations^1,2^.

PSOs can affect both adults and children, with minimal male preponderance^3^. Symptoms often include motor limitations, sphincter dysfunction, discomfort, and sensory deficits, depending on the anatomical location of the tumor^3^.

PSOs represent around 2% of all intramedullary (IM) spinal tumors, and 62 cases have been reported in the literature^3^. However, there are only eight cases of primary widespread spinal oligodendroglioma at this time and only one case of primary spinal cord oligodendroglioma with intracranial extension^1,2^.

Here, we present a case of a primary spinal oligodendroglioma with intracranial extension as the second case reported in the literature. This case report has been reported in line with the Surgical CAse REport (SCARE) criteria^4^.

## Case presentation

A 28-year-old right-handed female presented to the emergency department with left-sided hemiparesis, numbness, tingling, and urinary retention. The patient was found to be malaised with a high fever. Motor weakness started suddenly 10 days ago. However, her sensory symptoms developed gradually over 1 year, and 2 days before admission the patient developed ataxic gait and dysphagia. The patient had a past medical history of hypothyroidism which was being treated and she had no known food or drug allergies.

At examination, the patient showed left-sided motor weakness (a muscular strength score of 2/5 in the upper left limb and 3/5 in the lower left limb) with positive Babinski’s and negative Hoffman’s signs while the right side was normal.

The patient was admitted to the neurosurgical ward and laboratory tests were ordered. The results showed an elevated WBC count of 15.6×10^9^/l (Neutrophils 82.9%) and CRP 43.5 mg/dl. The urinalysis showed the presence of WBCs (18-20 HPF) in addition to epithelial cells (10-12 HPF), which confirms the presence of urinary infection most probably caused by the urinary retention that the patient presented with. Subsequently, she was put on broad-spectrum antibiotics.

**Figure 1:**
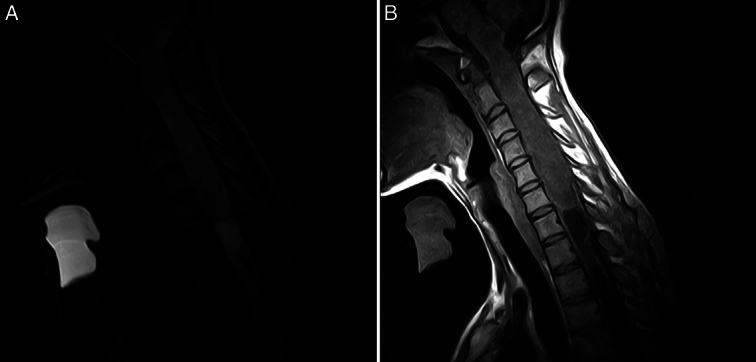
(Colored). A. MRI, sagittal section, T2: Shows a heterogeneous hyperintense lesion extending from C7 to the medulla. B. MRI, sagittal section, T1: Shows a heterogeneous Isointense lesion extending from C7 to the medulla with a hypointensity extending caudal to the mass (Syrinx).

MRI was ordered (Fig. 1), and it showed a widespread heterogeneous mass extending from the medulla to C7. The mass is 125 mm in length and 18×23 mm in diameter. The spinal canal is widened with no obvious skeletal damage and the mass is isointense in T1 and hyperintense on T2 with isointense spots.

After the administration of gadolinium, the MRI demonstrated a heterogeneously well-enhanced mass. The MRI also shows syringomyelia inferior to the mass.

Due to the patient’s deteriorating neurological condition, the team opted to perform urgent surgery to excise the tumor from her spinal cord. The CRP value was 20.3 when the surgery took place.

**Figure 2:**
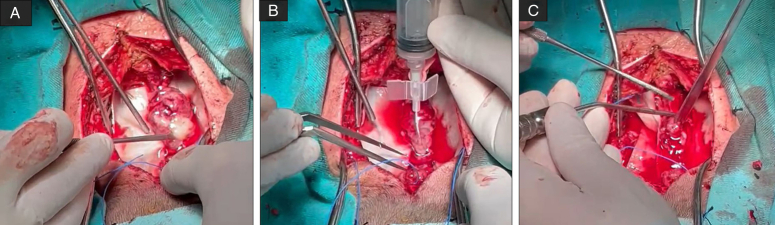
(Colored). A. surgical view, C2 to C7 bilateral laminectomy, the tumor is identified. B. surgical view, the tumor is dissected bluntly using saline. C. surgical view, the tumor is removed in a piecemeal fashion.

The surgery was performed with a posterior cervical midline incision with sufficient dissection of the musculature until the spinal column was reached. Then a C2 to C7 bilateral laminectomy (Fig. 2) was performed (staying away from C1 and the occiput), and the ligamentum flavum was incised sharply. The dura was visualized and incised sharply on the midline parallel to the posterior spinal fissure. The tumor was visualized and dissected bluntly using saline and the surgeon was able to remove it and specimens were sent to the pathology department. The dura was left open to prevent spinal compression and a Redon negative pressure drain was placed in the wound cavity. The drain was removed on postoperative day 5.

In the pathology department, gross examination showed several ill-defined tumor masses measuring 5×4 cm, gray-pink color, and soft consistency.

**Figure 3:**
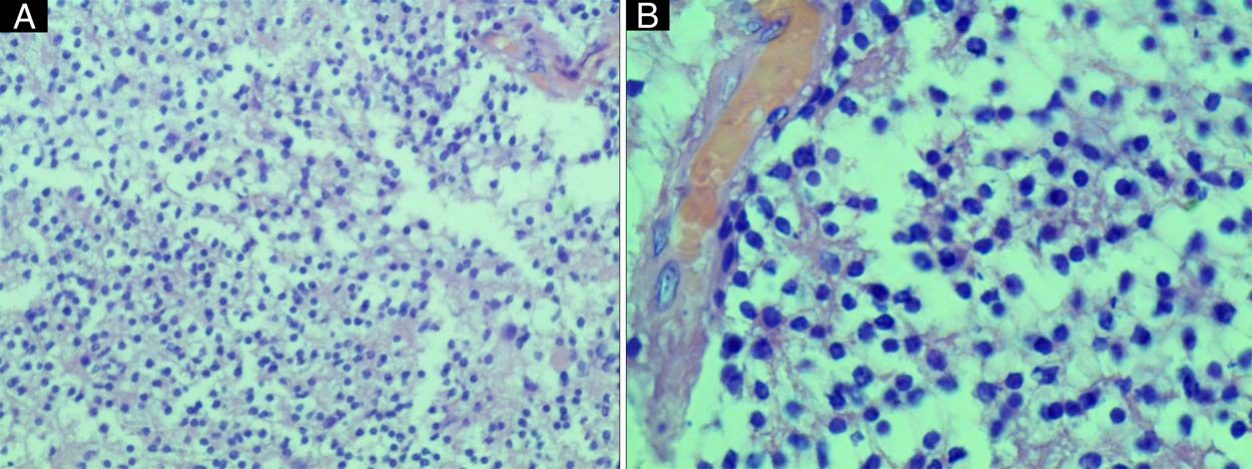
(Colored). A. Oligodendroglioma, diffuse proliferation of variable-sized glial cells mainly oligodendrocytes. (H&E×40). B. Oligodendroglioma, Neoplastic oligodendrocytes with central round or elongated hyperchromatic nuclei, clear cytoplasm, and occasional mitotic figures in addition to proliferation of small thin-walled blood vessels.

Microscopic examination (Fig. 3) showed diffuse proliferation of variable-sized neoplastic oligodendrocytes with large round or elongated hyperchromatic nuclei, clear cytoplasm, and occasional mitotic figures in addition to a proliferation of thin-walled blood vessels.

**Figure 4:**
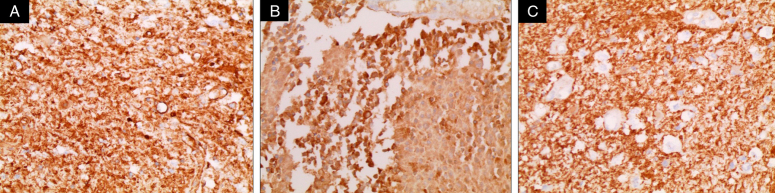
(Colored). A. Immunohistochemistry showing positivity for GFAP. B. Immunohistochemistry showing positivity for Vimentin. C. Immunohistochemistry showing positivity for S-100.

Immunohistochemistry (IHC) showed positivity for GFAP, S-100, and Vimentin. This confirmed the diagnosis of WHO grade II oligodendroglioma (Fig. 4). We note that the 1p19q deletion biomarker was not ordered because it was not available at our hospital.

**Figure 5:**
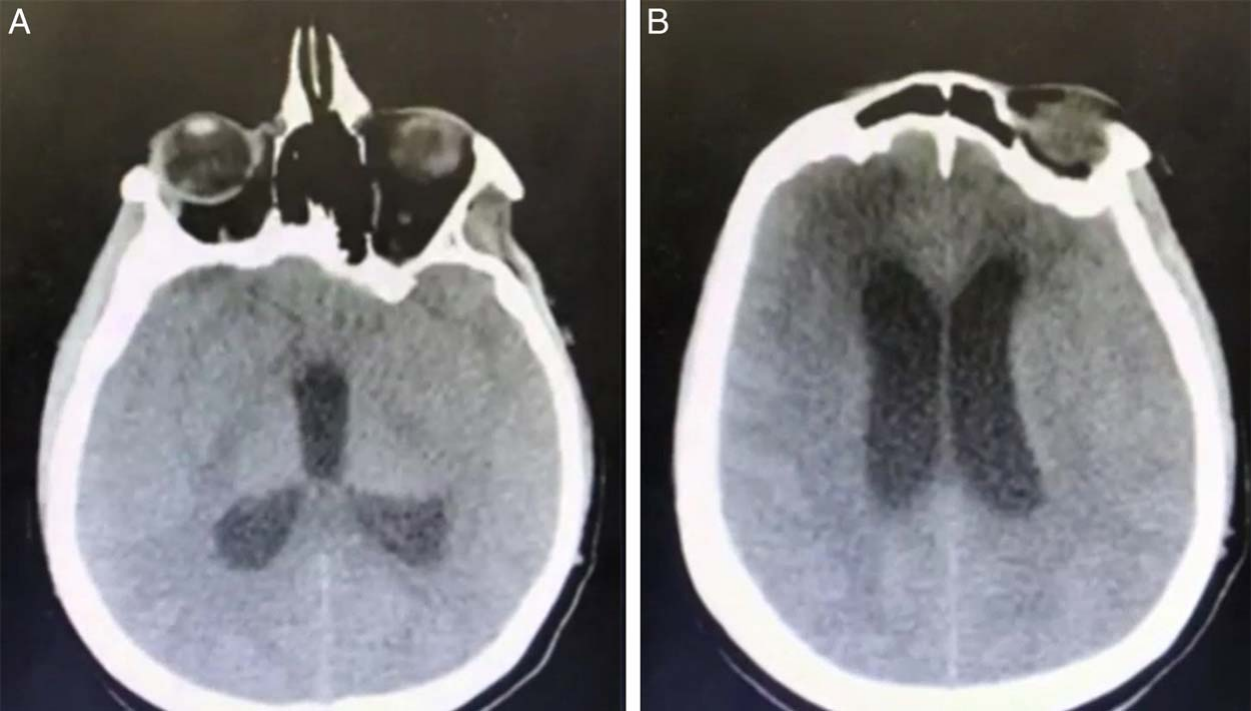
(Colored). A. CT, axial section, dilation of the 3rd ventricle indicating hydrocephalus. B. CT, axial section, dilation of the lateral ventricles indicating hydrocephalus.

Postoperatively the patient’s neurological condition improved significantly (a muscle strength score of 3/5 in the upper left limb and 4/5 in the lower left limb) and she was able to walk relatively well. She was due to follow up with an oncologist in seven days to be put on a radiotherapy treatment plan. However, on postoperative day 4, she complained of severe headaches that did not respond to analgesics. A CT scan was ordered, and it showed hydrocephalus (Fig. 5). Due to that the surgical team decided to put an extra dural shunt to treat the hydrocephalus.

Two days later her condition deteriorated, and she developed meningitis (positive neck stiffness and Kering). CSF samples were taken for culture, and she was put on a course of antibiotics (Levofloxacin, Ceftriaxone, and Vancomycin). The culture results showed pan-resistant Acinetobacter.

She was moved to the ICU for close monitoring. However, the patient did not respond to treatment and passed away 2 days after her admission to the ICU.

## Discussion

Oligodendrogliomas are most commonly detected in the cerebral hemispheres however, they may rarely be found in the spinal cord^1,2^. Also, oligodendrogliomas had the highest mean survival rate among other diffuse spinal gliomas^5^.

Symptoms of PSO are nonspecific depending mostly on the anatomical location of the tumor. These symptoms usually develop gradually over months to years^3^. Common symptoms include regional back pain and sensory disturbances while motor and sphincter defects usually occur later in the presentation (as shown in our case). Other less common symptoms include kyphoscoliosis and raised intracranial pressure^3^.

In 2017, a large case series by Hastruk *et al*.^3^studied every case of PSO from 1931 to 2016 bringing the total number of documented cases to 60.

During the period 2017 to 2023, we found two more cases of PSO by Cruz *et al*. and Eppy *et al*.^1,6^, which brings the total number of documented cases (without this case) to 62 and makes our case the 63rd recorded case of primary spinal oligodendroglioma.

We have searched the related literature extensively and only found a single case of a primary spinal oligodendroglioma with intracranial extension (Cruz *et al*.). Our case marks the second PSO in literature with intracranial extension. MRI is the primary imaging modality in spinal tumors^1,7^. Generally, PSOs are heterogeneous hypointense or isointense on T1 and hyperintense on T2 with moderate heterogeneous enhancement when gadolinium contrast is administered^1,7^. This heterogeneity is explained by the calcium accumulation, which is linked at the molecular level to 1p/19q deletion and these two features are pathognomonic to PSO^1,7^.

Pathological examination and subsequent immunohistochemistry provide the definitive diagnosis for PSO. Under the microscope WHO grade II oligodendroglioma (as in our case) shows high tumor cellularity with round to oval hyperchromatic nuclei, which are slightly larger than the nuclei of normal cells with occasional clumping of the chromatin material^6,8^. This classical histological appearance of oligodendroglioma is called the ‘honeycomb’ or ‘fried egg’^6^. Finally, immunohistochemical staining shows mild reactivity for GFAP^3^.

Prior studies have almost entirely been case reports which limit the ability to assess surgical outcomes in PSOs. The treatment choice we have found in the literature was mostly surgical excision^1,3,9^. However, there is not a clear guideline that indicates, which technique should be used. Staging of the procedure limits surgeon fatigue and ensures a safe and radical resection of the tumor, thus offering a chance for cure^10,11^. Epstien and Epstien^12^ found that surgical excision of multisegmented IM gliomas may be done with little to no neurological deficit. In our case, the patient underwent a single-stage C2 to C7 bilateral laminectomy. In most cases (after such an operation) we found that the patient’s neurological condition deteriorated rapidly. However, our patient’s neurological condition improved tremendously to the point that she was able to walk independently and her score on the muscular strength exam improved as mentioned above in the presentation.

The postresection radiation therapy (RT) has been linked to many complications including postradiation myelopathy and radiation-induced vertebral column deformities^3^. These adverse events are more frequent and more severe among pediatric patients which limits RT to older patients with subtotal resection or high-grade PSO^3^. In our case, the decision for RT was to continue the treatment after the subtotal resection. Sadly, due to the postoperative infection and the hydrocephalus the patient died a few days later.

## Conclusion

Primary spinal oligodendroglioma is an uncommon tumor that arises from oligodendrocytes in the spinal cord. Here, we presented the second known case in the literature that reports a primary spinal cord oligodendroglioma with intracranial extension. Single-stage laminectomy has shown good results and surgical resection was the key intervention to which the patient responded. These results matched what was stated in the literature that surgery is the primary mode of treatment in PSO patients.

## Ethical approval

Given the nature of the article, a case report, no ethical approval was required.

## Consent

Written informed consent was obtained from the patient for publication of this case report and accompanying images. A copy of the written consent is available for review by the Editor-in-Chief of this journal on request.

## Sources of funding

No funding was required.

## Author contributions

All authors contributed to this manuscript. All authors contributed to the writing and editing of this manuscript. G.B. and N.I.: collecting references, original draft writing, and editing; Y.E., T.D., and M.I.: original draft writing; K.K. and M.S.A.: reviewing and supervision; Z.A.: final reviewing and supervision.

## Conflicts of interest disclosure

The authors declares no conflicts of interest.

## Research registration unique identifying number (UIN)

Not needed.

## Guarantor

Prof. Zuheir Alshehabi.

## Data availability statement

The data is available on request.

## Provenance and peer review

Not commissioned, externally peer-reviewed.
